# Effect of cerebral dopamine neurotrophic factor on endogenous neural progenitor cell migration in a rat model of Parkinson's disease

**Published:** 2019-03-05

**Authors:** Ava Nasrolahi, Javad Mahmoudi, Mohammad Karimipour, Abolfazl Akbarzadeh, Saeed Sadigh-Eteghad, Roya Salehi, Fereshteh Farajdokht, Mehdi Farhoudi

**Affiliations:** 1 Neurosciences Research Center (NSRC), Tabriz University of Medical Sciences, Tabriz, Iran; 2 Department of Molecular Medicine, Faculty of Advanced Medical Sciences, Tabriz University of Medical Sciences, Tabriz, Iran; 3 Department of Applied Cell Sciences, Faculty of Advanced Medical Sciences, Tabriz University of Medical Sciences, Tabriz, Iran; 4 Department of Anatomical Sciences, Faculty of Medicine, Tabriz University of Medical Sciences, Tabriz, Iran; 5 Department of Medical Nanotechnology, Faculty of Advanced Medical Sciences, Tabriz University of Medical Sciences, Tabriz, Iran; 6 Department of Neuroscience, Faculty of Advanced Medical Sciences, Tabriz University of Medical Sciences, Tabriz, Iran

**Keywords:** 6-hydroxydopamine, CDNF, neural stem/progenitor cells, neurogenesis, neuronal migration, Parkinson's disease

## Abstract

This study investigated the ability of intra-subventricular zone (SVZ) administration of cerebral dopamine neurotrophic factor (CDNF) on neural progenitor cells (NPCs) attraction from the SVZ toward the 6-hydroxydopamine (6-OHDA)-lesioned striatum and improvement of motor dysfunctions in Parkinsonian rats. Male Wistar rats were assigned to four groups of the sham model (Sham), 6-OHDA-lesioned (OH), 6-OHDA-lesioned plus CDNF vehicle (OH+Vehicle), and 6-OHDA-lesioned plus CDNF (OH+CDNF). The animal model of Parkinson's disease (PD) was induced by unilateral intra-striatal infusion of 6-OHDA. Rats in the treatment groups received an intra-SVZ injection of CDNF or the vehicle of CDNF two weeks after PD model induction and were then subjected to the beam and bar tests on days 7, 14, and 21 after CDNF injection. Bromodeoxyuridine (BrdU) was intraperitoneally injected to label newly generated cells. Migration and proliferation of NPCs were assessed by BrdU/doublecortin (DCX) double immunofluorescence method on days 7, 14, and 21 after CDNF infusion. 6-OHDA in the OH group induced catalepsy and increased elapsed time in the beam test compared to the Sham group. However, administration of CDNF improved the motor performance and increased the number of DCX expressing neuroblasts in the SVZ as compared to the OH and OH+Vehicle groups. CDNF also enhanced cell proliferation and increased the number of migrated BrdU- and DCX-positive cells toward the lesioned striatum in the OH+CDNF group. These results suggest that CDNF enhances the proliferation and migration of neural stem cells (NSCs) toward the lesioned striatum accompanied by improvement of PD-induced motor dysfunctions.

## Introduction

Parkinson's disease (PD) is the second most common neurodegenerative condition associated with motor and non-motor symptoms, including akinesia, bradykinesia, muscle rigidity, resting tremor, dementia as well as gastrointestinal and sleep problems (Nasrolahi et al., 2018[25]). Death of nigrostriatal dopaminergic (DAergic) neurons is the major pathology of PD resulting in a significant decrease of striatal dopamine levels and onset of disease symptoms (O'Keeffe and Sullivan, 2018[27]). Current therapeutic options for PD have numerous limitations and only attenuate PD symptoms. Therefore, the search continues to discover more effective strategies for reversing the neurodegeneration of DAergic neurons or replacing the degenerated neurons by new ones.

Under normal condition, neurogenesis happens in the restricted regions of the adult mammalian brain such as subventricular zone (SVZ) and the subgranular zone (SGZ) (Ming and Song, 2011[23]). In the SVZ, activated neural progenitor cells (NPCs) are derived from neural stem cells (NSCs) and then migrate through the rostral migratory stream (RMS) toward the olfactory bulb where they differentiate into neurons (Lepousez et al., 2013[16]). However, pathological conditions such as PD negatively affect recruiting and mobilizing of NPCs towards the damaged target sites (Saha et al., 2012[33]; Zhao et al., 2008[48]). Although exogenous stem cell delivery is suggested as a solution to cope with this problem (Jiaming and Niu, 2015[13]), cell therapy has serious limitations including technical and ethical issues and may also increase the risk of tumorigenesis and immune response upon engraftment (Erdö et al., 2003[8]; Lindvall et al., 2004[18]; Liu and Huang, 2007[19]). Alternative strategies which provoke differentiation and migration of endogenous stem cells toward the lesion sites in the brain have lately received much attention (Fon et al., 2014[9]). In rodent models of PD, due to the particular anatomical location and proximity of the SVZ to the dopaminergic fibers, stem cells from this area are readily recruited to the lesion site. However, inadequate trophic support at this point may decrease the potential of NSCs for regeneration and replacement of the lost DAergic cells (Winner et al., 2008[45]). Growth factors may hold promising molecules to provide sufficient trophic support for NSCs at the lesion site.

Neurotrophic factors (NTFs) are small proteins that promote survival, differentiation, and maturation of neurons during development. Studies have established the potential therapeutic benefits of NTFs and proposed them as suitable candidates for the treatment of neurodegenerative conditions (Voutilainen et al., 2015[41]). Although glial-derived neurotrophic factor (GDNF) and neurturin (NRTN) are the most studied and promising NTFs in PD, their unintended effects and low clinical benefits direct attention to the other NTFs with a selective trophic activity on DAergic neurons (Bartus and Johnson Jr, 2017[5]; Huddleston and Factor, 2011[12]; Lang et al., 2006[15]). Cerebral dopamine neurotrophic factor (CDNF) is a member of the newly discovered family of evolutionarily conserved NTFs (Lindholm et al., 2007[17]). This factor exerts both neurorestorative and neuroprotective effects in several animal models mainly through its antioxidant, anti-apoptosis, and anti-inflammatory properties (Tang et al., 2017[38]). In addition, some specific features of CDNF, including its selectivity for DAergic neurons, having low side-effects and higher effectiveness at low or medium concentration than GDNF and BDNF making it a promising candidate for PD treatment (Nasrolahi et al., 2018[25]; Tang et al., 2017[38]). Moreover, CDNF mRNA has been detected in the embryonic midbrain, suggesting its crucial role in the development of DAergic neurons (Lindholm et al., 2007[17]). Interestingly, CDNF, in contrast to GDNF, can only exert protective effects on degenerated DAergic neurons and does not influence these neurons in the intact brain (Voutilainen et al., 2017[43]).

Doublecortin (DCX) is a microtubule-associated phosphoprotein, which is required for neural migration in the adult brain and expressed for the first 2 weeks after the birth of the neurons. 5'-bromo-2'-deoxyuridine (BrdU), an exogenous cell tracer, is used for detection of the neurogenesis within the adult central nervous system. DCX labels cytoplasm, while BrdU is a mitotic marker which labels the nucleus, thus they are ideal markers for double labeling of the neuroblasts (Wojtowicz and Kee, 2006[46]). In the present study, we investigated the effect of intra-SVZ administration of CDNF on the attraction of NPCs towards the lesion site in a 6-OHDA model of PD in rats.

## Material and Methods

### Animals

Seventy-two male Wistar rats (250-280 g) were obtained from the laboratory animal unit of Tabriz University of Medical Sciences (Tabriz, Iran). Animals were housed at a well-ventilated room with constant temperature (21±2 ºC) and under 12-h light/dark cycle with free access to food and tap water. The present study was approved by the Ethical Committee of Tabriz University of Medical Sciences (IR.TBZMED.REC.1395.1273) and all experimental procedures were conducted in accordance with the National Research Council Committee for the care and use of laboratory animals.

### Study design

One week following adaptation to a new condition, animals were randomly assigned to one of the following groups (n=18 in each group): sham model (Sham); 6-hydroxydopamine (6-OHDA)-lesioned (OH); 6-OHDA-lesioned plus CDNF vehicle (OH+Vehicle), and 6-OHDA-lesioned plus CDNF (OH+ CDNF). The Sham group underwent stereotaxic surgery procedure and only received the vehicle of 6-OHDA. The OH, OH+Vehicle, and OH+CDNF groups were subjected to the intra-striatal infusion of 6-OHDA. Two weeks following 6-OHDA injection, rats in the OH+Vehicle and OH+CDNF groups received a unilateral intra-SVZ injection of phosphate-buffered saline (PBS) and CDNF, respectively.

### 6-OHDA lesion and CDNF injection

Animals were anesthetized by inhalation of isofluorane gas (3 % administrated at 1.5 L/min), and then mounted onto a stereotactic frame with a nose-cone for anesthesia delivery. 6-OHDA hydrobromide (10 μg) was dissolved in 2 μL of 0.9 % ice-cold saline containing 0.02 % ascorbic acid (Tocris Bioscience, Bristol, UK) and injected to the right striatum at following coordinates (in mm) relative to bregma: AP +0.24, LM +3.6, DV -3.8 (Paxmos and Watson, 1982[28]). Also, rats were given intraperitoneal (i.p.) injection of 25 mg/ kg desipramine (Sigma Chemical Co., USA) 30 min prior to the 6-OHDA injection to prevent noradrenergic neuronal loss at the injection site (Mahmoudi et al., 2011[21]).

**Figure 1:**
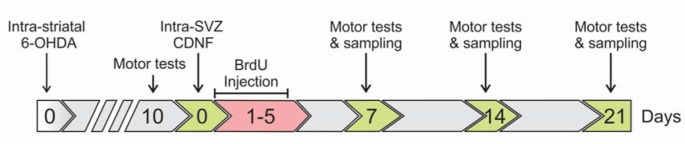
Scheme of the study design. 6-OHDA: 6-hydroxydopamine; CDNF: cerebral dopamine neurotrophic factor; BrdU: Bromodeoxyuridine; SVZ: subventricular zone

In the OH+CDNF group, 2 weeks following induction of PD, 10 µg of recombinant human CDNF (PeproTech, Rocky Hill, NJ, USA) in 4 µL of PBS was injected to the right SVZ at following coordinates: AP +0.24, LM +1.8, DV -3.8 (Paxmos and Watson, 1982[28]). Sham model rats were only injected with 2 μL vehicle of 6-OHDA (0.9 % saline containing 0.02 % (w/v) ascorbic acid) into the striatum. Furthermore, 6-OHDA-lesioned rats in the OH+Vehicle group received 4 µL of PBS (vehicle of CDNF) into the SVZ. The protocol of our experimental design is summarized in Figure 1(Fig. 1).

### 5-Bromo-2′-deoxyuridine (BrdU) injection

BrdU (Sigma Chemical Co, USA) was used to label the proliferating cells in the SVZ. For this purpose, 10 mg/ml BrdU was dissolved in 0.9 % NaCl and sterile-filtered at 0.22 µm. A day after injection of CDNF, 50 mg/kg of BrdU was injected intraperitoneally twice a day for 5 consecutive days. Subsequently, six rats from each group were sacrificed at different time intervals (7, 14, and 21 days) after injection of CDNF or vehicle. 

### Behavioral assessments

All animals were transferred to the testing room at least 30 min before the behavioral tests. Behavioral tests were performed once 10 days after 6-OHDA injection to confirm the induction of PD model as well as 7, 14, and 21 days after intra-SVZ injection of CDNF or vehicle to assess the possible effect of CDNF on the motor function of PD rats.

### Beam walking test

A 105 cm elevated beam with a flat surface of 4 cm width was used to assess both akinesia and bradykinesia behaviors in rats. The beam was suspended 80 cm above the floor using two tripod stands at both ends. A start line was marked by drawing a transversal line 20 cm from the one end of the beam and at the end point an enclosed black box containing home cage bedding material was placed as a motivation. In order to measure akinesia time, each rat was placed entirely behind the starting line facing the black box and the latency time to cross the starting line with all four feet within 1 min was recorded. Also, the total time spent to traverse the beam and reach to the black box was recorded as bradykinesia time. If an animal could not cross the total length of the beam within 2 min or fell down, the test was terminated and maximum time was recorded (Allbutt and Henderson, 2007[1]).

### Catalepsy test 

Catalepsy-like immobility was evaluated by placing the rat's forepaws on an elevated horizontal bar (9-cm height). The catalepsy latency was defined as the retention time in this immobile posture (Mahmoudi et al., 2011[21]) and recorded up to a cutoff time of 180 s.

### Immunofluorescence staining 

In order to find out whether CDNF promotes proliferation of NSCs and migration of NPCs in the SVZ, the number of BrdU- and doublecortin (DCX)-positive cells was measured using an immunofluorescence method. For this purpose, on days 7, 14, and 21 after CDNF injection (14, 28, and 35 days after infusion of 6-OHDA), animals were deeply anesthetized by i.p. injection of the ketamine and xylazine mixture (80 and 8 mg/kg, respectively) and were perfused transcardially with 200 ml of cold PBS, followed by 200 ml of 4 % paraformaldehyde (PFA; Sigma) (pH 7.4). Then, the brain was removed from the skull and post-fixed in 4 % PFA overnight and embedded in paraffin. Six series of 5 μm-thick coronal sections were cut between the bregma +0.36 and +0.12 mm, according to the atlas of Paxinos and Watson (1982[28]) with a microtome (Leica, Vienna, Austria) and mounted on poly-lysine-coated slides for immunofluorescence staining. In brief, the sections were deparaffinized and rehydrated through graded concentrations of alcohol and then washed in Tris-buffered saline (TBS; 0.1 M Tris-HCl, pH 7.4, and 0.9 % NaCl). Antigen retrieval was performed with incubation of sections in preheated 10 mM citrate buffer solution for 15 min at 100 ºC. After several washing in TBS, the sections were incubated in TBS 3 % goat serum 0.3 % Triton X (TBS11) for 30 min and then incubated for double-labeling in a mixture of primary antibodies, including rabbit anti-DCX (ab18723, Abcam, USA) and rat anti-BrdU (ab6326, Abcam, USA) at 4 ºC temperature. Primary and secondary antibodies were diluted in TBS containing 0.3 % Triton X-100 and 3 % goat serum. On the next day, after washing twice with TBS, the sections were incubated with a mixture of secondary antibodies conjugated with Alexa Fluor 488 or 594 (Invitrogen) in a humid and dark chamber at room temperature for 1 h. Subsequently, sections were washed twice with TBS, and nuclear counterstaining was performed with 40, 60-diamidino-2-phenylindole dihydrochloride hydrate (DAPI; Sigma-Aldrich) for 3 min. After washing twice with TBS, slides were mounted with glycerol buffer, coverslipped, and then visualized with a fluorescence microscope and digitally photographed (Zeiss, Axiophot, Germany). For counting the number of BrdU^+^ and DCX^+ ^cells, every six sections were selected and BrdU- and DCX-labeled cells were fully counted on each section. The number of BrdU^+^ cells and DCX-expressing neuroblasts in the striatum adjacent to the SVZ was estimated to assess the proliferation and migration patterns, respectively. Moreover, for investigation of co-expression of BrdU and DCX for neuronal phenotype, 100 BrdU-labeled cells per animal were analyzed and the ratio of BrdU-labeling cells co-expressing with DCX was determined. All counting procedures were conducted on a light/fluorescence microscope (Zeiss AxioImager M2, Germany).

### Statistical analysis

All statistical data were analyzed using GraphPad Prism 7.0 software, USA. Results were expressed as mean ± standard error of the mean (SEM) and were analyzed using two-way ANOVA followed by Tukey's post-hoc test. *P*-value < 0.05 was considered statistically significant. 

## Results

The first session of motor tests was performed 10 days after injection of 6-OHDA and the rats which displayed motor dysfunctions compared to the Sham animals were considered as Parkinsonian rats (data are not shown).

### CDNF attenuates catalepsy-like behavior in the bar test

**Figure 2:**
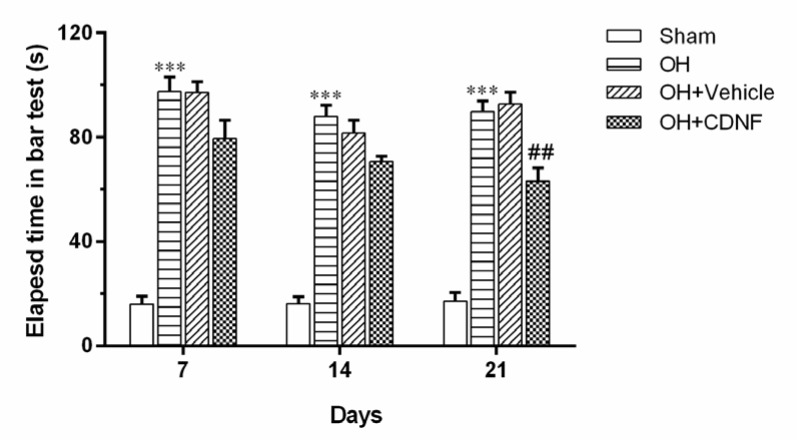
Comparison of the elapsed time in the bar test in different groups on days 7, 14, and 21 after injection of CDNF or vehicle. Each bar represents the mean ± SEM of elapsed time (s); n=6 per each group; ***P< 0.001 vs. Sham group; ^##^*P*< 0.01 vs. OH+Vehicle group. (OH: 6-OHDA; CDNF: cerebral dopamine neurotrophic factor)

The elapsed time was assessed in the bar test on days 7, 14, and 21 after injection of CDNF. As depicted in Figure 2(Fig. 2), the results from the two-way ANOVA of elapsed time in the bar test using group and day as factors demonstrated the main effect of group (F (3, 60) = 188.6, *P*<0.001), day (F (2, 60) = 4.051, *P*<0.05), and group × day interaction (F (6, 60) = 1.310, *P*>0.05). Intergroup analysis showed that elapsed time was significantly increased in the OH group on days 7, 14, and 21 (*P*<0.001 for all days) compared to the Sham group. However, infusion of CDNF significantly (*P*<0.01) decreased elapsed time on day 21 as compared to the OH+Vehicle group. 

### CDNF attenuates akinesia and bradykinesia in the beam test

**Figure 3:**
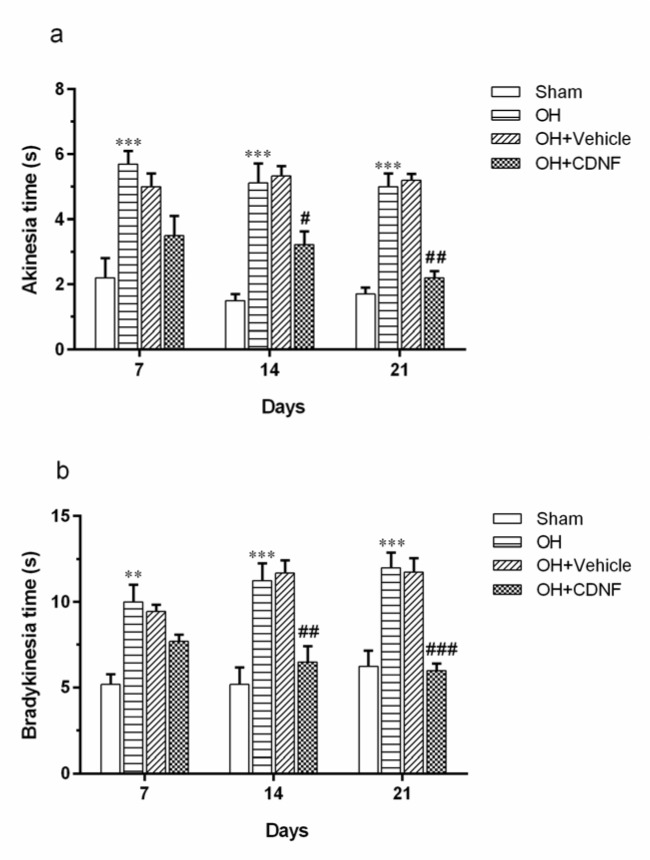
The results of the beam test in Sham, OH, OH+Vehicle, and OH+CDNF groups. (a) Comparison of the latency to begin crossing and (b) total time to cross the beam between rats on days 7, 14, and 21 after injection of CDNF or vehicle. Each bar represents the mean ± SEM (n=6 rats for each group); **P< 0.01, ***P< 0.001 vs. Sham group ^#^*P*< 0.05, ^##^*P*< 0.01, ^###^*P*< 0.001 vs. OH+Vehicle group. (OH: 6-OHDA; CDNF: cerebral dopamine neurotrophic factor)

The results from the two-way ANOVA of the latency to begin crossing the beam (akinesia time) in the beam test using group and day as factors revealed the main effect of group (F (3, 60) = 53.10, *P*<0.001), day (F (2, 60) = 2.01, *P*>0.05), and group × day interaction (F (6, 60) = 0.8855, *P*>0.05). Intergroup analysis showed that akinesia time was significantly increased in the OH group on days 7, 14, and 21 (*P*<0.001 for all days) compared to the Sham group. However, intra-SVZ infusion of CDNF significantly decreased akinesia time on days 14 (*P*<0.05) and 21 (*P*<0.01) as compared to the OH+Vehicle group (Figure 3a(Fig. 3)).

Moreover, the results from the two-way ANOVA of total time spent to cross the beam (bradykinesia) in the beam test using group and day as factors demonstrated the main effect of group (F (3, 60) = 39.43, *P*<0.001), day (F (2, 60) = 1.36, *P*>0.05), and group × day interaction (F (6, 60) = 1.62, *P*>0.05). Intergroup analysis showed that bradykinesia time was significantly increased in the OH group on days 7 (*P*<0.01), 14 (*P*<0.001), and 21 (*P*<0.001) compared to the Sham group. However, intra-SVZ infusion of CDNF significantly decreased bradykinesia time on days 14 (*P*<0.01) and 21 (*P*<0.001) as compared to the OH+Vehicle group (Figure 3b(Fig. 3)).

### CDNF increases proliferation of NPCs in the SVZ

**Figure 4:**
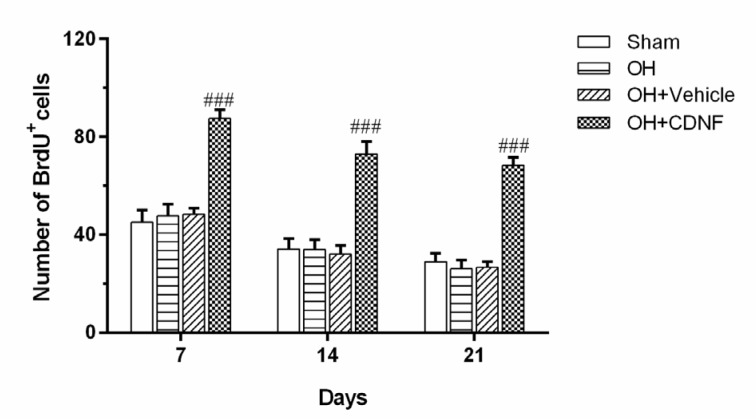
Quantification of BrdU^+^ cells in the SVZ 7, 14, and 21 days after the CDNF injection. Each bar represents the mean ± SEM (n=6 rats for each group); ^##^*P*< 0.01, ^###^*P*< 0.001 vs. OH+Vehicle group. (OH: 6-OHDA; CDNF: cerebral dopamine neurotrophic factor)

**Figure 5:**
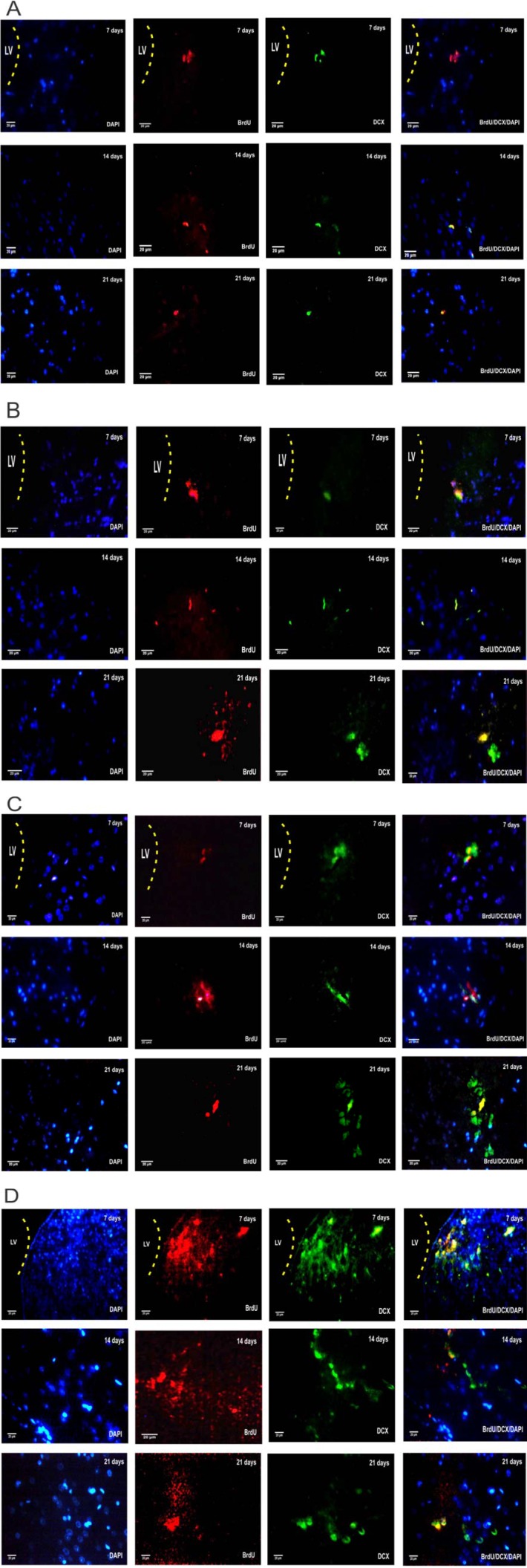
CDNF stimulates the proliferation, survival, and migration of neural stem cells (NSCs) and neural progenitor cells (NPCs) of SVZ in 6-OHDA-lesioned rats. Immunostaining of BrdU/DCX-positive cells in the SVZ 7, 14, and 21 days after the CDNF injection. A: Sham group. B: OH (6-OHDA-lesioned) group. C: OH+Vehicle group (6-OHDA-lesioned plus CDNF vehicle). D: OH+CDNF group (6-OHDA-lesioned plus CDNF). Sections were double-labeled for BrdU (red), DCX (green) and DAPI (blue).

In order to investigate the effect of CDNF and its vehicle on the proliferation and survival of NSCs, we evaluated the incorporation of BrdU, as a proliferation marker in different groups. The results from the two-way ANOVA of the number of BrdU-positive cells using group and day as factors showed the main effect of group (F (3, 60)= 79.25, *P*<0.001), day (F (2, 60) = 26.44, *P*<0.001), and group × day interaction (F (6, 60) = 0.9893, *P*>0.05). Intergroup analysis showed that intra-SVZ infusion of CDNF significantly increased BrdU+ cells on days 7, 14, and 21 (*P*<0.001 for all days) as compared to the OH+Vehicle group. This suggests that CDNF enhanced the proliferation of the NSCs and NPCs in the SVZ (Figure 4(Fig. 4) and Figure 5(Fig. 5)).

In order to assess the survival rate of BrdU-labeled cells, we determined the ratio of BrdU^+^ cells at 3^rd^ week with labeled cells at 1^st^ week after the last intra-SVZ injection of CDNF. The results showed that the survival rates of BrdU+ cells in the Sham, OH, OH+Vehicle, and OH+CDNF groups were as 64.19 %, 54.47 %, 55.34 %, and 78.11 %, respectively. These results indicated that the number of BrdU^+^ cells significantly decreased in the OH and OH+Vehicle groups at 3^rd^ week relative to the number of labeled cells at the 1^st^ week (*P*<0.05 for both). However, this decrease in the OH+CDNF group was not significant. Therefore, it might be inferred that CDNF could also improve the survival rate of the newly generated neuronal cells. 

### CDNF promotes the migration of DCX^+^ neuroblasts toward the striatum

**Figure 6:**
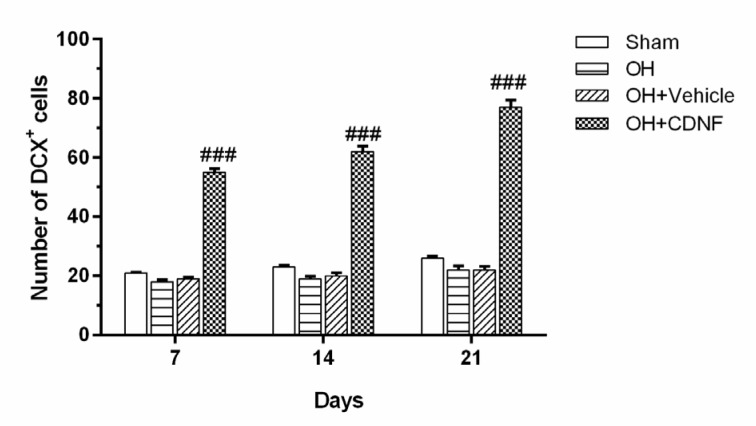
Quantification of DCX^+^ cells in the striatum adjacent to the SVZ 7, 14 and 21 days after the CDNF injection. Each bar represents the mean ± SEM (n=6 rats for each group); ^###^*P*< 0.001 vs. OH+Vehicle group. (OH: 6-OHDA; CDNF: cerebral dopamine neurotrophic factor)

DCX is a marker of migrating immature neurons which is expressed in NPCs in the early stage of neural differentiation during adult neurogenesis. We investigated the influence of intra-SVZ infusion of CDNF on the number of DCX+ neuroblasts within the lesioned striatum adjacent to the injection site. The results from the two-way ANOVA of the number of DCX-positive cells using group and day as factors showed the main effect of group (F (3, 60) = 947.3, *P*<0.001), day (F (2, 60)= 49.83, *P*<0.001), and group × day interaction (F (6, 60)= 14.15, *P*<0.001). Intergroup analysis showed that intra-SVZ infusion of CDNF significantly increased the number of DCX-expressing neuroblasts in the striatum on days 7, 14, and 21 (P<0.001 for all days) as compared to the OH+Vehicle group (Figure 6(Fig. 6) and Figure 5(Fig. 5)). These results suggest the migration of DCX-expressing neuroblasts from the SVZ toward the striatum in the OH+CDNF group. 

**Figure 7:**
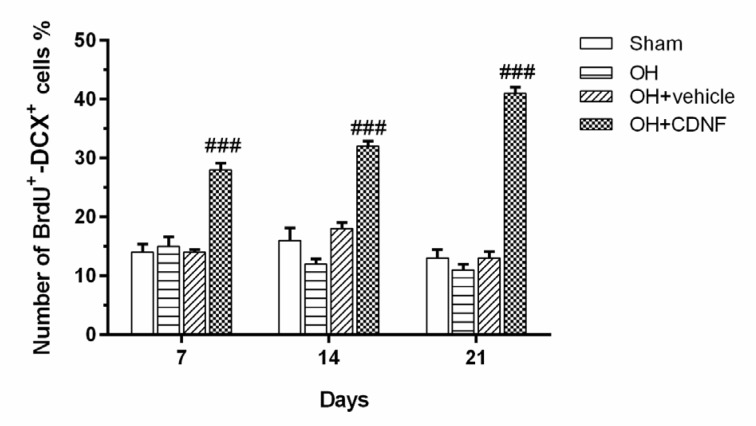
Quantification of BrdU- DCX-positive cells in the lesioned striatum adjacent to the SVZ 7, 14 and 21 days after the CDNF injection. Each bar represents the mean ± SEM (n=6 rats for each group); ^###^*P*< 0.001 vs. OH+Vehicle group. (OH: 6-OHDA; CDNF: cerebral dopamine neurotrophic factor)

The results from the two-way ANOVA of the number of double-labeling of BrdU/DCX-positive cells using group and day as factors showed the main effect of group (F (3, 60) = 188.3, *P*<0.001), day (F (2, 60) = 2.62, *P*<0.05), and group × day interaction (F (6, 60) = 11.55, *P*<0.001). Intergroup analysis showed that intra-SVZ infusion of CDNF significantly increased the number of DCX-expressing neuroblasts in the striatum on days 7, 14, and 21 (*P*<0.001 for all days) as compared to the OH+Vehicle group. However, there was no significant difference between the other groups (Figure 7(Fig. 7) and Figure 5(Fig. 5)). Considering these findings overall suggest that CDNF could enhance the neural migration and drives the NSCs fate to neural lineage.

See also the Supplementary data.

## Discussion

The results of the present study showed that motor dysfunctions were gradually improved within 3 weeks following CDNF treatment in the 6-OHDA-lesioned rats. Moreover, this treatment not only increases the proliferation and survival of NSCs and NPCs in the SVZ but also provokes migration toward the lesioned-striatum of the Parkinsonian rats. 

DAergic neurons in the striatum are prone to neurotoxic effects of the 6-OHDA. Previous reports have shown that intra-striatal injection of 6-OHDA results in loss of DAergic neurons approximately a day after injection (Quiroga-Varela et al., 2017[31]). 6-OHDA is taken up preferentially by DAergic neurons accumulating in the cytosol and inducing degeneration of these cells. Also, 6-OHDA induces neurotoxicity through inhibition of mitochondrial respiratory chain by formation of free radicals and oxidative damage, which is then followed by the death of DAergic neurons and subsequent decline in the striatal dopamine levels within 2 to 4 days (Schober, 2004[36]). These cellular and neurochemical changes are generally associated with motor dysfunctions such as akinesia (Luo et al., 2016[20]), bradykinesia (Sanders and Jaeger, 2016[35]), and catalepsy (Prajapati et al., 2017[30]). In accordance with previous reports, our results also revealed that intra-striatal injection of 6-OHDA prolonged the duration of catalepsy, akinesia, and bradykinesia.

Previous studies have shown that NTFs including neurturin (NTRN), GDNF, and CDNF improve rotational behavior following administration of 6-OHDA in animal models (Oiwa et al., 2002[26]; Sajadi et al., 2006[34]; Voutilainen et al., 2011[42]). Moreover, different strategies for intra-striatal delivery of CDNF using adeno-associated virus 2 (AAV2) and mesenchymal stem cells (MSCs) which express CDNF lead to improvement of behavioral impairments in 6-OHDA-lesioned rats (Bäck et al., 2013[4]; Jiaming and Niu, 2015[13]). In this study, for the first time, we have shown that intra-SVZ administration of CDNF improves motor dysfunctions in the 6-OHDA-lesioned striatum. In contrast to dopamine replacement therapy focusing on modifying dopamine levels, current strategies attempt to reverse disease progression using neurorestorative agents. Moreover, some NTFs such as GDNF, NRTN, and CDNF are able to rescue DAergic neurons probably through the stabilization of tyrosine hydroxylase expression in these neurons leading to improvement of behavioral impairments (Nasrolahi et al., 2018[25]). Although CDNF has a neuroprotective effect on DAergic neurons, the exact mechanism of action of CDNF has not yet been elucidated.

Adult neurogenesis is defined as the born of new neurons from endogenous NSCs in the adult brain, consisting of the proliferation, survival, and differentiation processes (Kumaria and Tolias, 2012[14]). As age advances, these processes of the NSCs progressively are declined (Tocharus et al., 2014[39]). Moreover, the recruitment and mobilization of NPCs towards the degenerating site are decreased in age-related disorders such as PD (Saha et al., 2012[33]; Zhao et al., 2008[48]). Given the regulatory role of dopamine in endogenous adult neurogenesis (Höglinger et al., 2004[11]), loss of DAergic neurons and the subsequent depletion of dopamine in the substantia nigra may diminish cell proliferation in the SVZ (Borta and Höglinger, 2007[6]). Therefore, enhancing the proliferation, survival, and recruitment of endogenous NSCs in the PD brain can be a step towards PD treatment in the future.

Ongoing studies suggest the potential role of NSCs for replacement of lost DAergic neurons in PD. Although exogenous stem cell delivery is suggested as a solution to tackle this problem (Jiaming and Niu, 2015[13]), it has serious limitations and may also increase the risk of tumor and immune response (Erdö et al., 2003[8]; Lindvall et al., 2004[18]; Liu and Huang, 2007[19]). However, the promotion of neurogenesis of endogenous NSCs in the adult brain could repair the nigrostriatal DAergic system and may provide a self-repair mechanism without having above limitations. 

In the SVZ of rodents, more than 30,000 neuroblasts per day migrate through the RMS toward the olfactory bulb where they differentiate to new olfactory interneurons (Alvarez-Buylla et al., 2001[2]). NSCs in the adult SVZ and SGZ might be utilized for replacement of lost neurons in neurodegenerative disorders (Fon et al., 2014[9]). Owing to the vicinity of SVZ to the nigrostriatal DAergic fibers, NSCs from this area can be easily recruited to the lesion site (Winner et al., 2008[45]). Under normal conditions, multiple factors including NTFs regulate the generation, survival, migration, and differentiation of newborn neurons (Hagg, 2005[10]). Alteration of these brain factor levels, which occurs in neurodegenerative diseases such as PD, may affect adult neurogenesis in the SVZ (Borta and Höglinger, 2007[6]). Previous studies have also demonstrated that growth factors can facilitate the recruitment of NPCs and neurogenesis in response to lesions (Nakatomi et al., 2002[24]; Sun et al., 2002[37]; Winner et al., 2008[45]).

Endogenous and exogenous delivery of NTFs including ciliary neurotrophic factor (Emsley and Hagg, 2003[7]), GDNF (Yuan et al., 2013[47]), and BDNF (Wei et al., 2015[44]) have been shown to enhance neurogenesis. Furthermore, the neuroprotective effects of NTFs such as GDNF, BDNF, NTRN, and CDNF on the nigrostriatal DAergic system has been reported in animal models of PD (Nasrolahi et al., 2018[25]; Voutilainen et al., 2015[41]). *In vivo *and* in vitro *studies have also reported that the direct infusion of CDNF proteins or CDNF-engineered vectors significantly protect and reverse the loss of DAergic neurons in PD models (Lindholm et al., 2007[17]; Mei and Niu, 2015[22]; Ren et al., 2013[32]). In this study, we investigated the effect of CDNF as a potent neurotrophic factor on neuroblast migration from the SVZ toward the 6-OHDA-lesioned striatum as a possible cell replacement strategy in this region. BrdU was injected intraperitoneally 11 days after the induction of PD for assessment of the proliferation and survival of NSCs and NPCs, and then its incorporation was analyzed. Our results revealed an increase in the number of BrdU^+^ cells after the infusion of CDNF, indicating that CDNF enhances the proliferation of the NSCs and NPCs in the SVZ. 

DCX is a microtubule-associated protein which stabilizes the microtubules in the immature neurons during migration. This protein expresses for approximately 3 weeks after the birth of newborn neurons and serves as a reliable marker for migrated neurons (Bachstetter et al., 2011[3]). Our data also showed a significant increase in the number of DCX^+^ neuroblasts within the lesioned striatum reflecting a radial migration of neuroblasts from the SVZ into the adjacent striatum in the CDNF-treated group. 

Mesencephalic astrocyte-derived neurotrophic factor (MANF) and CDNF belong to the evolutionarily conserved neurotrophic factor family which have protective effects on DAergic neurons in animal models of PD (Lindholm et al., 2007[17]; Petrova et al., 2003[29]). Moreover, a recent study has introduced MANF as a critical regulator of neurite growth, neuronal migration, and development of mammalian cortex. MANF deletion has been shown to reduce neuronal migration and impair neurite outgrowth confirming its essential role in neurogenesis under normal conditions (Tseng et al., 2017[40]). Therefore, it is likely that CDNF, through a similar mechanism as MANF, increases neurogenesis. Taken together, this study provides evidence that CDNF may be considered as an important factor to stimulate the migration of NSCs from the SVZ to the lesioned-striatum.

Our findings showed that intra-SVZ infusion of CDNF enhances adult neurogenesis and reverses the motor dysfunctions induced by 6-OHDA in a rat model of PD and suggests that CDNF can be a promising component for treatment of PD symptoms. However, it is not clear whether differentiation of immature DCX^+ ^neuroblasts to DAergic neurons within the striatum or retrograde movement of CDNF from the SVZ to the striatum leads to an improvement of motor dysfunction. In the present study, we did not measure the number of tyrosine hydroxylase-positive cells. Therefore, future studies which measure the expression of tyrosine hydroxylase and dopamine levels in the lesion site are proposed as the next step to confirm the role of CDNF-induced newborn DAergic neurons restoring the dopamine levels and motor dysfunction. These studies may be helpful in recognizing the exact mechanism of CDNF in the migration of NSCs and their differentiation to DAergic neurons in PD.

## Notes

Javad Mahmoudi and Mehdi Farhoudi (Neurosciences Research Center (NSRC), Tabriz University of Medical Sciences, Tabriz, Iran, Postal code: 5166614756; Tel: +984133351284, E-mail: Farhoudi_m@yahoo.com) contributed equally as corresponding authors.

## Acknowledgement

The authors would like to acknowledge the staff of Neurosciences Research Center (NSRC) for their support in this project.

## Funding source

This study was supported by a grant (No. 95/4-5/5) from Neurosciences Research Center (NSRC), Tabriz University of Medical Sciences, Tabriz, Iran. 

## Disclosure statement

The authors would like to acknowledge the staff of Neurosciences Research Center (NSRC) for their support in this project.

## Supplementary Material

Supplementary data
