# Supplementary material for: Effect of cerebral dopamine neurotrophic factor on endogenous neural progenitor cell migration in a rat model of Parkinson's disease
Source: EXCLI J. 2019 Mar 5;18:139–53. (PMC6449669)
Supplement: Supplementary data [file EXCLI-18-139-s-001.pdf]

EXCLI Journal 2019;18:139-153 – ISSN 1611-2156
Received: December 01, 2018, accepted: February 02, 2019, published: March 05, 2019

Supplementary data to

EFFECT OF CEREBRAL DOPAMINE NEUROTROPHIC FACTOR ON ENDOGENOUS NEURAL PROGENITOR CELL MIGRATION IN A RAT MODEL OF PARKINSON'S DISEASE

Ava Nasrolahi[1,2], Javad Mahmoudi[*1], Mohammad Karimipour[3,4], Abolfazl Akbarzadeh[5], Saeed Sadigh-Eteghad[1], Roya Salehi[5], Fereshteh Farajdokht[1], Mehdi Farhoudi[*1,6]

1 Neurosciences Research Center (NSRC), Tabriz University of Medical Sciences, Tabriz, Iran
2 Department of Molecular Medicine, Faculty of Advanced Medical Sciences, Tabriz University of Medical Sciences, Tabriz, Iran
3 Department of Applied Cell Sciences, Faculty of Advanced Medical Sciences, Tabriz University of Medical Sciences, Tabriz, Iran
4 Department of Anatomical Sciences, Faculty of Medicine, Tabriz University of Medical Sciences, Tabriz, Iran
5 Department of Medical Nanotechnology, Faculty of Advanced Medical Sciences, Tabriz University of Medical Sciences, Tabriz, Iran
6 Department of Neuroscience, Faculty of Advanced Medical Sciences, Tabriz University of Medical Sciences, Tabriz, Iran

* Corresponding authors: Professor Mehdi Farhoudi and Dr. Javad Mahmoudi, Neurosciences Research Center (NSRC), Tabriz University of Medical Sciences, Tabriz, Iran, Postal code: 5166614756; Tel: +984133351284, E-mail: Farhoudi_m@yahoo.com; Mahmoudi2044@yahoo.com

http://dx.doi.org/10.17179/excli2018-1959

This is an Open Access article distributed unter the terms of the Creative Commons Attribution License (http://creativecommons.org/licenses/by/4.0).

EXCLI Journal 2019;18:139-153 – ISSN 1611-2156
Received: December 01, 2018, accepted: February 02, 2019, published: March 05, 2019

The raw data of the elapsed time (s) in the bar test in different groups on days 7, 14, and 21 after injection of CDNF or vehicle. n=6 per each group (OH: 6-OHDA; CDNF: cerebral dopamine neurotrophic factor). Each line corresponds to one rat.

**Elapsed time in the bar test 7**				
Animal number	Sham	OH	OH+Vehicle	OH+CDNF
Rat no. 1	10	101	96	74
Rat no. 2	9	119.57	110	80
Rat no. 3	19.01	78.45	108	66.4
Rat no. 4	28	89	89	58
Rat no. 5	10	100	85.5	102.6
Rat no. 6	20.09	97	95	96

**Elapsed time in the bar test 14**				
Animal number	Sham	OH	OH+Vehicle	OH+CDNF
Rat no. 1	24.48	86	80.5	66
Rat no. 2	13	96	96.7	76.3
Rat no. 3	9.6	105.2	77	74.5
Rat no. 4	9.02	78	60.3	66.2
Rat no. 5	19	80	84.5	75.25
Rat no. 6	22.4	82.8	90	66.25

**Elapsed time in the bar test 21**				
Animal number	Sham	OH	OH+Vehicle	OH+CDNF
Rat no. 1	27.2	97.9	110.2	76.75
Rat no. 2	10	91	86	60.5
Rat no. 3	25	105	99	50.25
Rat no. 4	9.8	81.1	94	65
Rat no. 5	10.5	78	90	78
Rat no. 6	21	85.5	77.3	49

The raw data of Table 3(a). The latency to begin crossing the beam (Akinesia time(S)) in different groups on days 7, 14, and 21 after injection of CDNF or vehicle. n=6 per each group (OH: 6-OHDA; CDNF: cerebral dopamine neurotrophic factor). Each line corresponds to one rat.

**Akinesia 7**				
Animal number	Sham	OH	OH+Vehicle	OH+CDNF
Rat no. 1	1.85	5.3	5	5
Rat no. 2	2	4.4	3.8	1.75
Rat no. 3	1.2	6	4	2
Rat no. 4	1.8	5	5	4
Rat no. 5	1.2	7.1	6.2	5.25
Rat no. 6	5.15	6.4	6	3

**Akinesia 14**				
Animal number	Sham	OH	OH+Vehicle	OH+CDNF
Rat no. 1	2	6.85	6.1	4.5
Rat no. 2	1	6	5	4.35
Rat no. 3	1.6	3.59	4.45	3
Rat no. 4	0.9	6.1	6.45	2.55
Rat no. 5	1.45	3.1	5	2
Rat no. 6	2.1	5.1	5	3

**Akinesia 21**				
Animal number	Sham	OH	OH+Vehicle	OH+CDNF
Rat no. 1	1.9	5	5	2.5
Rat no. 2	1	6.2	5.65	1.8
Rat no. 3	1.3	4	4.8	1.56
Rat no. 4	2	5	4.8	2.85
Rat no. 5	1.6	3.9	5	2.5
Rat no. 6	2.4	6.1	6	2

EXCLI Journal 2019;18:139-153 – ISSN 1611-2156
Received: December 01, 2018, accepted: February 02, 2019, published: March 05, 2019

The raw data of the total time to cross the beam (Bradykinesia time (S)) in different groups on days 7, 14, and 21 after injection of CDNF or vehicle. n=6 per each group (OH: 6-OHDA; CDNF: cerebral dopamine neurotrophic factor). Each line corresponds to one rat.

**Bradykinesia7**				
Animal number	Sham	OH	OH+Vehicle	OH+CDNF
Rat no. 1	4.45	8	9.8	8.5
Rat no. 2	3.55	7.5	8.9	7
Rat no. 3	7.74	10	10.1	6.8
Rat no. 4	6	12	10.8	8.5
Rat no. 5	5	13.9	8	8.8
Rat no. 6	4.5	9	9.1	6.6

**Bradykinesia14**				
Animal number	Sham	OH	OH+Vehicle	OH+CDNF
Rat no. 1	7	10	11	8
Rat no. 2	5	10	12.85	4
Rat no. 3	3	12.5	13	7.65
Rat no. 4	3	7.98	10	6
Rat no. 5	9.2	12	9.35	9.35
Rat no. 6	4	15	14	4

**Bradykinesia21**				
Animal number	Sham	OH	OH+Vehicle	OH+CDNF
Rat no. 1	6	13	12	6
Rat no. 2	4.7	12	11	5
Rat no. 3	4.8	10	13.5	7
Rat no. 4	8.7	14	10	6
Rat no. 5	9.25	14.25	14.5	7.2
Rat no. 6	4.05	8.75	9.5	4.8

The raw data of the number of BrdU+ cells in six series of coronal sections per rats in different groups on days 7, 14, and 21 after injection of CDNF or vehicle. n=6 per each group (OH: 6-OHDA; CDNF: cerebral dopamine neurotrophic factor). Each line corresponds to one rat.

**BrdU7**				
Animal number	Sham	OH	OH+Vehicle	OH+CDNF
Rat no. 1	39.08	32.8	39	79
Rat no. 2	32.18	52.7	48	99
Rat no. 3	60.04	58	50	91
Rat no. 4	47.2	49	48	82
Rat no. 5	33.72	60	58.8	96.05
Rat no. 6	58.9	34	46.2	77.95

**BrdU14**				
Animal number	Sham	OH	OH+Vehicle	OH+CDNF
Rat no. 1	47.09	29	21	58
Rat no. 2	21.22	47	39.62	88.03
Rat no. 3	45	44.11	41	79
Rat no. 4	37	36	37	66.97
Rat no. 5	25.91	23	32	61
Rat no. 6	29	24.89	22.88	85

**BrdU21**				
Animal number	Sham	OH	OH+Vehicle	OH+CDNF
Rat no. 1	18.25	17	26	59
Rat no. 2	21.05	37.95	35.1	74
Rat no. 3	39.85	34.75	30.5	66
Rat no. 4	37	28	28	68.1
Rat no. 5	28.95	19	21	61.95
Rat no. 6	31.95	20	19.9	81.05

The raw data of the number of DCX+ cells in six series of coronal sections per rats in different groups on days 7, 14, and 21 after injection of CDNF or vehicle. n=6 per each group (OH: 6-OHDA; CDNF: cerebral dopamine neurotrophic factor). Each line corresponds to one rat.

**DCX7**				
Animal number	Sham	OH	OH+Vehicle	OH+CDNF
Rat no. 1	21	18	20	54
Rat no. 2	20.7	20.9	19.4	56.4
Rat no. 3	20.85	16	21	60
Rat no. 4	21.95	16.3	17.5	55
Rat no. 5	20.5	19.8	19	53
Rat no. 6	21	17	17.1	51.6

**DCX14**				
Animal number	Sham	OH	OH+Vehicle	OH+CDNF
Rat no. 1	23	19.75	22	67
Rat no. 2	25.85	18	21	66.15
Rat no. 3	22.15	17.25	23.45	64
Rat no. 4	23	23	19.35	56
Rat no. 5	22	17	18.2	56.85
Rat no. 6	22	19	16	62

**DCX21**				
Animal number	Sham	OH	OH+Vehicle	OH+CDNF
Rat no. 1	27	24.95	23	86.55
Rat no. 2	26	23	26.75	81
Rat no. 3	23.1	17.05	18.25	71
Rat no. 4	25	19	20	74
Rat no. 5	27	22	21	72.45
Rat no. 6	27.9	26	23	77

The raw data of the number of BrdU+/DCX + cells in six series of coronal sections per rats in different groups on days 7, 14, and 21 after injection of CDNF or vehicle. n=6 per each group (OH: 6-OHDA; CDNF: cerebral dopamine neurotrophic factor). Each line corresponds to one rat.

**BrdU/DCX7**				
Animal number	Sham	OH	OH+Vehicle	OH+CDNF
Rat no. 1	17	17	16	31.5
Rat no. 2	9	16.95	14.36	28.75
Rat no. 3	18	21	14	30
Rat no. 4	14	12	14	26
Rat no. 5	11	10.05	12.64	27.75
Rat no. 6	15	13	13	24

**BrdU/DCX14**				
Animal number	Sham	OH	OH+Vehicle	OH+CDNF
Rat no. 1	16.5	12	15	33
Rat no. 2	24	13.4	15	33
Rat no. 3	13.5	10.6	18	31
Rat no. 4	10	9	20	31
Rat no. 5	20	12	21	29
Rat no. 6	12	15	19	35

**BrdU/DCX21**				
Animal number	Sham	OH	OH+Vehicle	OH+CDNF
Rat no. 1	16	13.5	15	45
Rat no. 2	18	13.45	17	43.15
Rat no. 3	9	8.05	11	38.85
Rat no. 4	13	9	12.55	38
Rat no. 5	9	12	9.45	41
Rat no. 6	13	10	13	40
